# Tailoring p-Type Behavior in ZnO Quantum Dots through Enhanced Sol–Gel Synthesis: Mechanistic Insights into Zinc Vacancies

**DOI:** 10.1021/acs.jpclett.3c03519

**Published:** 2024-02-07

**Authors:** Abdullah Kahraman, Etienne Socie, Maryam Nazari, Dimitrios Kazazis, Merve Buldu-Akturk, Victoria Kabanova, Elisa Biasin, Grigory Smolentsev, Daniel Grolimund, Emre Erdem, Jacques E. Moser, Andrea Cannizzo, Camila Bacellar, Christopher Milne

**Affiliations:** ○ Paul Scherrer Institut, CH-5232 Villigen PSI, Switzerland; ■ École polytechnique fédérale de Lausanne (EPFL), Rte Cantonale, 1015 Lausanne, Switzerland; § Institute of Applied Physics, University of Bern, Sidlerstrasse 5, 3012 Bern, Switzerland; ∥ Faculty of Engineering and Natural Sciences, Sabanci University, Tuzla 34956 Istanbul, Turkey; ⊥ Physical Sciences Division, Pacific Northwest National Laboratory, Richland, Washington 99352, United States; □ European XFEL GmbH, 22869 Schenefeld, Germany

## Abstract

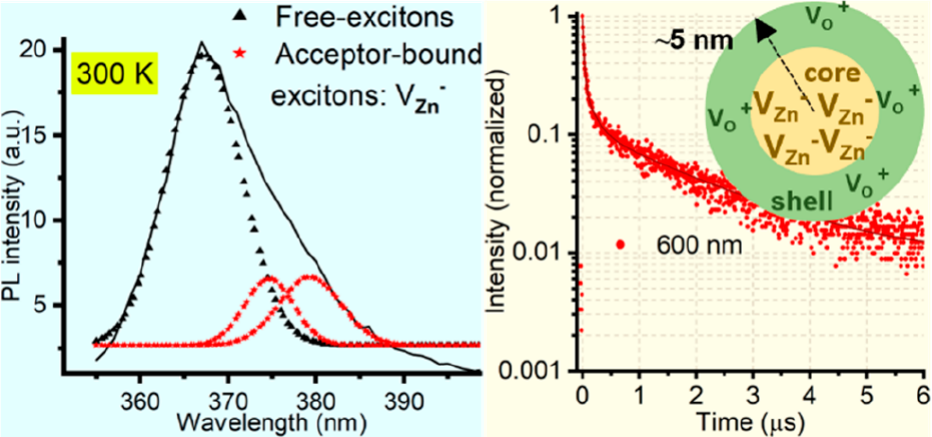

The synthesis and control of properties of p-type ZnO is crucial for a variety of optoelectronic and spintronic applications; however, it remains challenging due to the control of intrinsic midgap (defect) states. In this study, we demonstrate a synthetic route to yield colloidal ZnO quantum dots (QD) via an enhanced sol–gel process that effectively eliminates the residual intermediate reaction molecules, which would otherwise weaken the excitonic emission. This process supports the creation of ZnO with p-type properties or compensation of inherited n-type defects, primarily due to zinc vacancies under oxygen-rich conditions. The in-depth analysis of carrier recombination in the midgap across several time scales reveals microsecond carrier lifetimes at room temperature which are expected to occur via zinc vacancy defects, supporting the promoted p-type character of the synthesized ZnO QDs.

The enduring appeal of zinc oxide (ZnO) as a semiconductor stems from its abundance, low-cost, nontoxicity, direct band gap excitation (approximately 3.3 eV), high exciton binding energy (about 60 meV at room temperature), and the ease with which it can be synthesized from bulk to nano sizes.^1−3^ Being an inherently n-type semiconductor due to intrinsic donor defect states,^1^ ZnO finds extensive applications as heterojunction material in photovoltaics,^4^ photocatalysis,^5^ optical neural interfaces,^6^ spintronics,^7^ and more.^2,3^ However, challenges in the fabrication of p-type ZnO have hindered the practical application of devices based solely on ZnO,^8,9^ specifically as a light-emitting diode (LED), despite its higher exciton binding energy compared to GaN which is the leading material for LEDs.^10^ Within the diverse array of nanomaterials, quantum dots (QD) stand out due to their unique tunability of electronic energy levels via quantum confinement, making them exceptional materials for optoelectronic devices such as QD-LEDs,^11^ dye-sensitized solar cells based on ZnO QDs,^12^ sensors utilizing ZnO QDs,^13^ and a variety of other applications.^14^ The size of the QDs can also aid in controlling the structural features: studies focusing on defect phenomena of ZnO nanocrystals have suggested that the concentration of the intrinsic defects and their position can be tuned by merely changing the size of the nanocrystal so p-type ZnO QDs can be achieved.^2^

Despite its importance, the reproducible and high-quality fabrication of p-type ZnO remains challenging due to strong self-compensation effects from native defects: intrinsic donor defects, i.e., oxygen vacancies (V_o_), which spontaneously form and compensate the intentionally introduced acceptors.^1,8^ Nevertheless, under oxygen-rich conditions, p-type conductivity has been achieved in ZnO films^15,16^ and single crystals.^17^ Both experimental^8,18,19^ and theoretical^20,21^ studies propose the zinc vacancy (V_Zn_) in oxygen-rich stoichiometry as the origin of the p-type character of ZnO. Still, the nature of defects in ZnO is an open question: The type (*n* or *p*) and concentration of defects, also known as midgap “trap” states, vary with synthesis/fabrication methods and size, and this complicates the understanding of the band gap structure. For instance, almost all native defects of ZnO have been assigned as the origin of green emission, which has been extensively reviewed.^1,2,22^ However, the intricate defect mechanisms can be better understood for QD ZnO with respect to its bulk or nano version due to their distinct features: ultraviolet (UV) emission was observed to become more intense from bulk to nanoparticles (NPs) (10–100 nm) to QDs (2–10 nm) due to larger surface area and quantum confinement.^23^ Probing the carrier dynamics through the UV emission and ground-state depletion can give us crucial information on the competition of photoexcited electron relaxation between inter gap (UV) and midgap (visible emission) states.^22^

The photoluminescence characteristic of ZnO, which depends on these defects, can be effectively tuned using a “sol–gel” method by simply adjusting the reaction temperature and time.^16,24,25^ In this work, we produced ZnO QDs suspended in ethanol with a uniform size of approximately 4.5 nm by modifying the synthesis parameters of a sol–gel method previously reported^26^ and introducing new synthesis processes. The oxygen-rich stoichiometry of these QDs is demonstrated through energy dispersive X-ray spectroscopy (EDX) while their distinct QD features are shown through sharp exciton ultraviolet peaks in the photoluminescence emission and excitation spectra at room temperature. The compensation of n-type defects and promoting p-type characteristics has been analyzed by X-ray absorption near edge spectroscopy (XANES) of the Zn K-edge and through the core–shell structure by electron paramagnetic resonance (EPR).

We further investigate the complex mechanism of excited carrier trapping through midgap defect states by using time-resolved spectroscopy. Carrier relaxation to these trap states spans time scales from femtoseconds (fs) to picoseconds (ps), and ultimate relaxation to the valence band can take nanoseconds (ns).^27,28^ However, since optical time-resolved spectroscopy does not provide element-specific information, it remains challenging to resolve the specific defect-related mechanisms that drive carrier relaxation in ZnO, which was reported to span more than 8 orders of magnitude in time.^22^ The use of QDs in oxygen-rich conditions, however, simplifies the assignment of defect-related carrier dynamics since it has been theoretically shown that oxygen-rich conditions substantiate the presence of acceptor V_Zn_ states.^18,19,23^

*Synthesis and Characterization of ZnO Quantum Dots*. We synthesized colloidal ZnO quantum dots (QDs) using the sol–gel method originally described by Ullah et al.,^26^ albeit with modified reaction parameters and additional steps. The sol–gel process includes four crucial stages: hydrolysis, particle formation via monomer nucleation, growth, and aging. It is possible to fine-tune the final particle size and distribution during the aging phase.^24−26,29^ A detailed description of the modifications made to the established method is provided in the Supporting Information. Additional modifications starting from the final step of the reference method,^26^ shown in Scheme 1a, will be discussed here after presenting the key reactions.

**Scheme 1:**
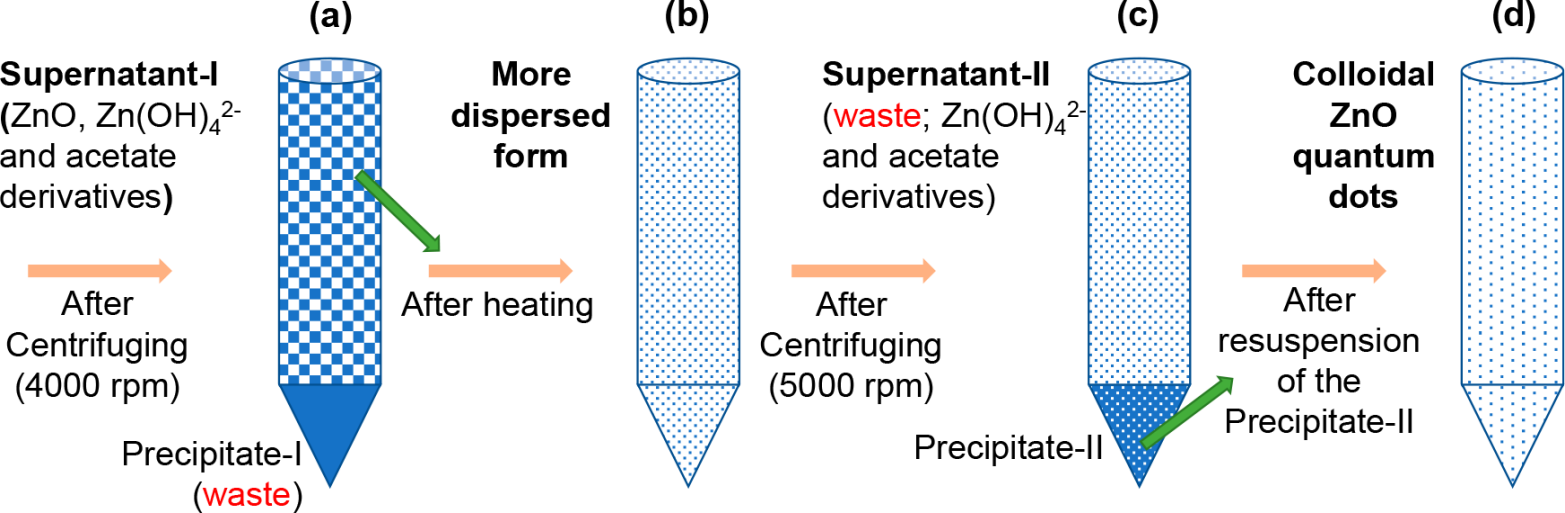
Synthesis Steps of ZnO QDs (a) After first centrifuging based on the reference sol–gel method.^26^ (b) Heating of supernatant-I in uncapped bottle at 68 °C for 2 h. (c) Centrifuging at 5000 rpm after controlled evaporation of ethanol eliminates the soluble reaction products; (d) Colloidal ZnO QDs are formed after minimizing the excess reaction products.

The sol–gel process includes four crucial stages: hydrolysis, particle formation via monomer nucleation, growth, and aging. The key reactions, beginning from hydrolysis during NaOH addition, are as follows:
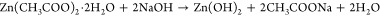
1After dissolving the zinc-acetate dihydrate (Zn(CH_3_COO)_2_·2H_2_O) in ethanol, hydrolysis occurs (eq 1). The Zn(OH)_2_ further reacts with the water molecules to form Zn(OH)_4_^2–^ and hydrogen ions:
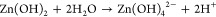
2In alkaline conditions, transformation of Zn(OH)_4_^2–^ into ZnO and the reverse reaction is possible:^29^
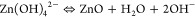
3Supernatant-I (Scheme 1a) likely retains ZnO nanoparticles, residual Zn(OH)_4_^2–^, and ethanolic zinc acetate byproducts, remaining in suspension due to omitted purification steps.^30−32^ Zn(OH)_2_, being insoluble, was expected to settle out along with other insoluble byproducts and larger ZnO particles,^31−33^ shown by precipitate-I (Scheme 1a). We initiated our modification process by heating (Scheme 1b), during which approximately 20% of the ethanol evaporated from uncapped bottles at a temperature of 68 °C for 2 h. This temperature helped us evaporate the ethanol in a controlled way. Increased ZnO concentration improved turbidity, an indicator of enhanced suspendability.^34^ This indicates a more uniform distribution of nanoparticles in the solution, reducing their tendency to settle quickly. This can happen when the particles are well-dispersed and stabilized in the solvent.^34^ It is important to note that ZnO can dissolve to a degree in alkaline conditions, forming Zn(OH)_4_^2^^–^. Consequently, some ZnO nanoparticles may convert to Zn(OH)_4_^2–^ at this stage, while any Zn(OH)_4_^2–^ remaining from Supernatant-I may reform ZnO through eq 3. The evaporation of ethanol in Supernatant-I might promote the dissolution of residual Zn(OH)_2_, potentially enhancing the formation of Zn(OH)_4_^2^^–^ as indicated in eq 2. This process could lead to the formation of a thin passivation layer or shell structure of ZnO, similar to what has been previously reported in the literature.^35^ Aiming to remove soluble Zn(OH)_4_^2–^, we conducted a second, more vigorous centrifugation (Scheme 1c) at 1000 rpm, higher than the initial centrifugation. This was done to ensure the collection of all ZnO particles, as the second centrifugation aimed to purify the colloidal system of Zn(OH)_4_^2^^–^ and zinc-acetate byproducts, in contrast to the first centrifugation, which focused on precipitating out larger particles. The precipitate-II was then “washed” and resuspended in ethanol to produce the final colloidal ZnO product (Scheme 1d). Using XANES, we were able to assess the reaction products resulting more accurately from further modifications.

Figure 1a presents the Zn K-edge spectrum of the final ZnO product, marked with green triangles, which is consistent with the expected spectrum for ZnO as referenced in previous studies.^36,37^ We observe specific electronic transitions from the zinc 1s core level to the 4p orbitals at about 9664 eV, appearing as a shoulder on the rising edge, and the primary peak at about 9667 eV, known as the “white line”, corresponding to the lowest and higher unoccupied orbitals, respectively. Features at energies above these points relate to the ionization potential and are influenced by multiple scattering events indicative of the structure.^37^ The Zn K-edge spectrum of Supernatant-I, shown with red dots, appears to be a composite of spectra from the precursor (zinc acetate dihydrate in ethanol, depicted by black circles), Supernatant-II (blue triangles), and the final product (green triangles). The observed 0.6 eV shift in the energy between the spectra of Supernatant-II and the precursor may originate from the excess Zn(OH)_4_^2^^–^ in Supernatant-II, which is attributable to its solubility.^29,30,32^ Since both zinc acetate and Zn(OH)_4_^2^^–^ display tetrahedral coordination akin to ZnO, the variation in peak positions between the precursor and Supernatant-II arises from the different chemical environments and the resulting shielding effects on the zinc 1s electrons.^30,38^ This supports our hypothesis that we successfully removed not only residual zinc acetate but also Zn(OH)_4_^2–^ by resuspending Precipitate-II as outlined in Scheme 1c. The varying ligand structures and coordination environments lead to shifts in the rising edges between ZnO and other zinc species, with further details provided below.

**Figure 1:**
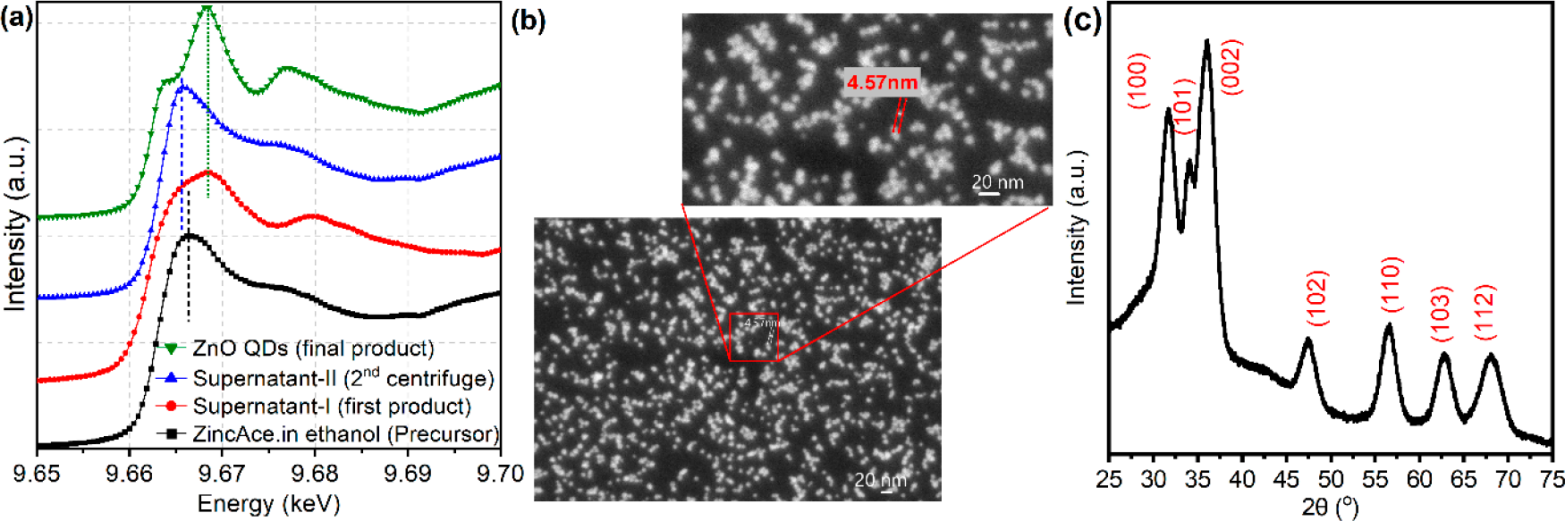
Structural analysis of ZnO QDs. (a) XANES spectra at the Zn K-edge of various synthesis stages and the final ZnO QDs product were measured in ethanol. Supernatant-I represents the initial product referenced from previous studies, while Supernatant-II results from subsequent procedural enhancements. The zinc acetate precursor’s spectrum is included for comparison (marked as black squares). Blue and black dashed vertical lines mark the main Zn K-edge peaks of Supernatant-II and the precursor, respectively. A green dashed vertical line indicates the peak maxima for the ZnO QDs and Supernatant-I. (b) SEM image of ZnO QDs dispersed on a Si wafer after ethanol evaporation, captured at a 5 kV acceleration voltage, showing an average dot diameter of 4.5 nm. (c) XRD pattern of ZnO QDs exhibiting the wurtzite structure, measured prior to resuspension in ethanol.

Additionally, the Zn K-edge XANES spectrum offers insights into the presence of oxygen vacancies (V_o_) in ZnO.^39^ The d^10^ configuration of Zn^2+^ typically precludes a pre-edge feature in ZnO’s spectrum. However, oxygen vacancies can modify the local zinc oxidation state and perturb the 3d orbitals. The absence of oxygen creates a new peak in the pre-edge area, more prominent with the removal of a second oxygen atom.^37,39,40^ Such a pre-edge peak, expected below 9660 eV,^37^ was notably absent in our spectra, suggesting a low concentration of V_O_ in our ZnO QDs. Moreover, we showed that the large number of Zn vacancies are likely to lead to the increase in the strength of the 1s → 4p in our previous study.^37^ This can be clearly seen by comparing the XANES spectra before and after the elimination of Zn(OH)_4_^2–^ (Figure 1a). Efficient decomposition of Zn(OH)_4_^2^^–^, as described earlier, would result in both less residual zinc and potentially increased oxygen incorporation into the ZnO structure. These observations suggest that compensating n-type defects (i.e., oxygen vacancies) could promote a p-type character (i.e., zinc vacancies). The development of this hypothesis will be further refined with subsequent results.

Scanning electron microscopy (SEM) provides insights into the size, shape, aggregation state, and surface morphology of the QDs. Figure 1b showcases a uniform distribution of ZnO QDs, with an average size of 4.5 ± 1.0 nm. X-ray diffraction (XRD) is essential for analyzing the crystallographic structure of materials. This is exemplified in Figure 1c for our ZnO QDs, which exhibit the characteristic peaks indicative of the wurtzite phase.^35^ The broadening of XRD peaks, as crystallite sizes diminish from bulk to nanoscale, is quantitatively explained by the Scherrer equation.^41^ Considering the (002) plane, the particle size was determined to be 4.16 nm, as detailed in the Supporting Information. It should be noted, however, that the size of the crystallite domain might not directly equate to the actual particle size, especially in the context of polycrystalline formation. Nevertheless, direct imaging using the SEM technique we employed corroborates this measurement.^41^

*Assessment of Defects by EPR and EDX Measurements*. Electron paramagnetic resonance (EPR) is a spectroscopic method frequently used to analyze materials containing unpaired electrons such as radicals, transition metal ions, and certain types of defect centers. EPR’s remarkable sensitivity to unpaired electrons and their immediate environment allows for the assessment of their electronic structure, bonding, and intermolecular interactions. Consequently, EPR is skillful at detecting trace paramagnetic species in predominantly diamagnetic or nonmagnetic materials.^2,42−44^ For ZnO, paramagnetic species might arise from an oxygen vacancy (V_O_), a zinc vacancy (V_Zn_), and Zn and O interstitials.^34^Figure 2a shows the X-band (9.64 GHz) EPR of ZnO QDs measured at room temperature. The first derivative of the absorption spectrum shows a resonance at a magnetic field *B* = 3444 G, corresponding to a calculated g-factor of 2.00 from the formula *hv* = *B*μ_Bohr_*g*. Further details on this calculation can be found in the Supporting Information. In nanoscale ZnO, distinct EPR signals with g-factor values around 1.96 and 2.00 are typically expected.^2^ We previously showed that the transition from bulk to nanoscale dimensions induces a decrease in the intensity of the *g* ≈ 1.96 signal which even vanishes in the QD size, coupled with an enhancement of the *g* ≈ 2.00 signal.^42−46^ This phenomenon is best explained by a core–shell model,^42,45^ where the *g* ≈ 1.96 signal is attributed to defects within the lattice core, while the *g* ≈ 2.00 signal originates from defects located at the surface. Shifting from bulk to nanoscale dimensions results in a diminished intensity of the core signal, accompanied by an increase in the surface defect signal.^42−44,46^ This is because, as ZnO transitions from bulk to nano form, the ratio of surface atoms to volume atoms increases. Among these defect species, V_O_ and V_Zn_ are widely recognized to have the lowest formation energy in ZnO.^20^ Drawing from our prior research, we propose that the weak *g* ≈ 2.00 signal seen in Figure 2a mainly arises from a small quantity of singly ionized V_O_^+^ localized at the surface, whereas the core signal (*g* = 1.96), observed in both bulk and nanocrystal ZnO and linked to V_Zn_^–^,^42−46^ becomes undetectable owing to the increased electron confinement at surface defects with decreasing particle size.

**Figure 2:**
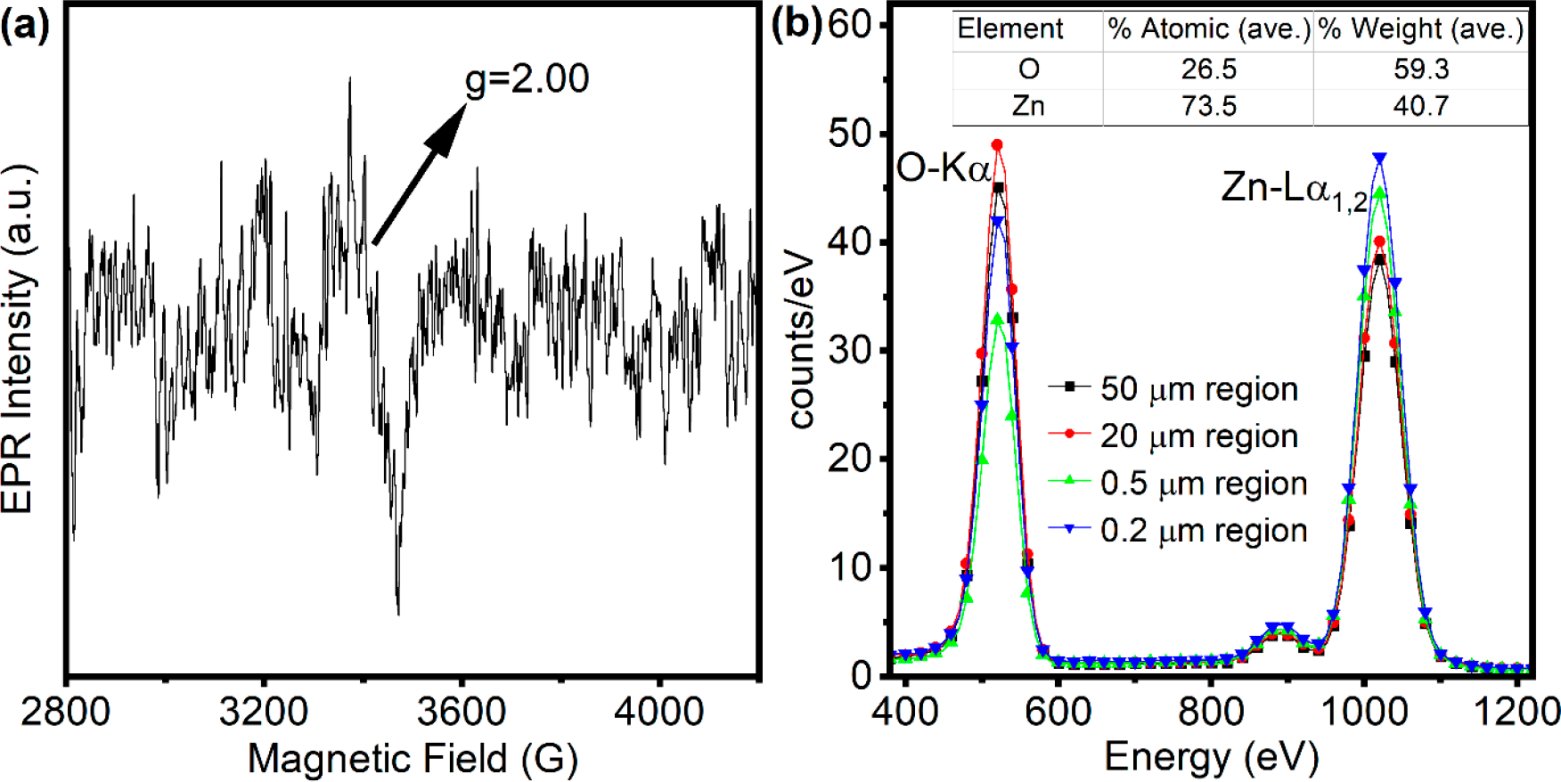
(a) X-band (9.64 GHz) EPR measurements at room temperature with 1 G modulation amplitude, 2 mW microwave power, and 50 scans of (b) EDX of ZnO QDs obtained on Si wafer upon ethanol evaporation (cps/eV vs Energy (eV)). The table shows the average stoichiometry from the measurements on mapping areas of 0.2, 0.5, 20, and 50 μm.

Energy-dispersive X-ray spectroscopy (EDX) was employed to analyze the chemical composition of the ZnO QDs. The EDX spectrum is depicted in Figure 2b, presenting the counts per second per electronvolt (cps/eV) plotted against energy. This was achieved by dividing the total counts by both the live time and the energy resolution, set at 0.01 eV. To validate the elemental composition of our sample, we conducted measurements on mapping areas of 0.2, 0.5, 20, and 50 μm. These measurements revealed an atomic composition of roughly 59.3 ± 0.1% oxygen (O) and 40.7 ± 0.1% zinc (Zn) by integrating the O 1s Kα and Zn Lα_1,2_ peaks. Given the pivotal role of this measurement in our work, we conducted EDX analyses on an additional sample, examining three distinct regions, all of which confirmed the oxygen-rich (53 ± 0.1% O or higher) stoichiometry (not shown here). This suggests that our ZnO QDs are rich in zinc vacancies (V_Zn_) due to their lowest formation energy among other possible defects in this composition, for instance, oxygen interstitials.^20,21^

*Photoluminescence Studies*. The photoluminescence spectrum of the ZnO QDs, upon exposure to 350 nm light, features a prominent emission peak at 367 nm, along with a green emission band between 425 and 650 nm (Figure 3a). The 367 nm peak is due to excitonic recombination, and its sharpness is due to the significant quantum confinement effect displayed by ZnO QDs,^23^ a phenomenon that has been similarly observed in studies for bulk and nanocrystalline ZnO at only low temperatures −100 K^22^ and at 2 K,^47^ respectively; at higher temperatures, electron–lattice interaction distorts the emission which is minimized in QDs due to well-confined (discrete) energy levels. As shown in Figure 3a inset, this main peak displays a low-energy shoulder. This peak can be deconvoluted into three Gaussian components centered at 367, 375, and 380 nm (Supporting Information). The 367 nm peak is ascribed to free-exciton FX_UV_ (an electron in the conduction band and a hole in the valence band) recombination. Meanwhile, the peaks of 375 and 380 nm can be attributed to the recombination of defect-bound exciton AX_UV_ (localization of excitons at acceptor defect site near the valence band maximum) which form prior to the generation of free excitons by trapping free carriers at defect sites.^18,22,47,48^ In addition to these peaks, Figure 3a shows a broad visible emission band spanning nearly 1 eV (435–650 nm) with a maximum intensity at 530 nm. The observed emission band arises from the combination of photogenerated electron relaxations, including direct transitions from the conduction band to deep acceptor states, sequential transitions from the conduction band through shallow donors to deep acceptors, transitions from deep donor states to shallow acceptor states, and transitions from deep donors to the valence band, among others.^16,22^ Such processes, which we refer to collectively as AX_VIS_, are characterized by dominant transitions depicted in Scheme 2. Comparable transitions, labeled as donor–acceptor-pair emission in another study, attribute donor states to oxygen vacancies (V_O_) and acceptor states to zinc vacancies (V_Zn_) in oxygen-rich ZnO.^16,49^

**Figure 3:**
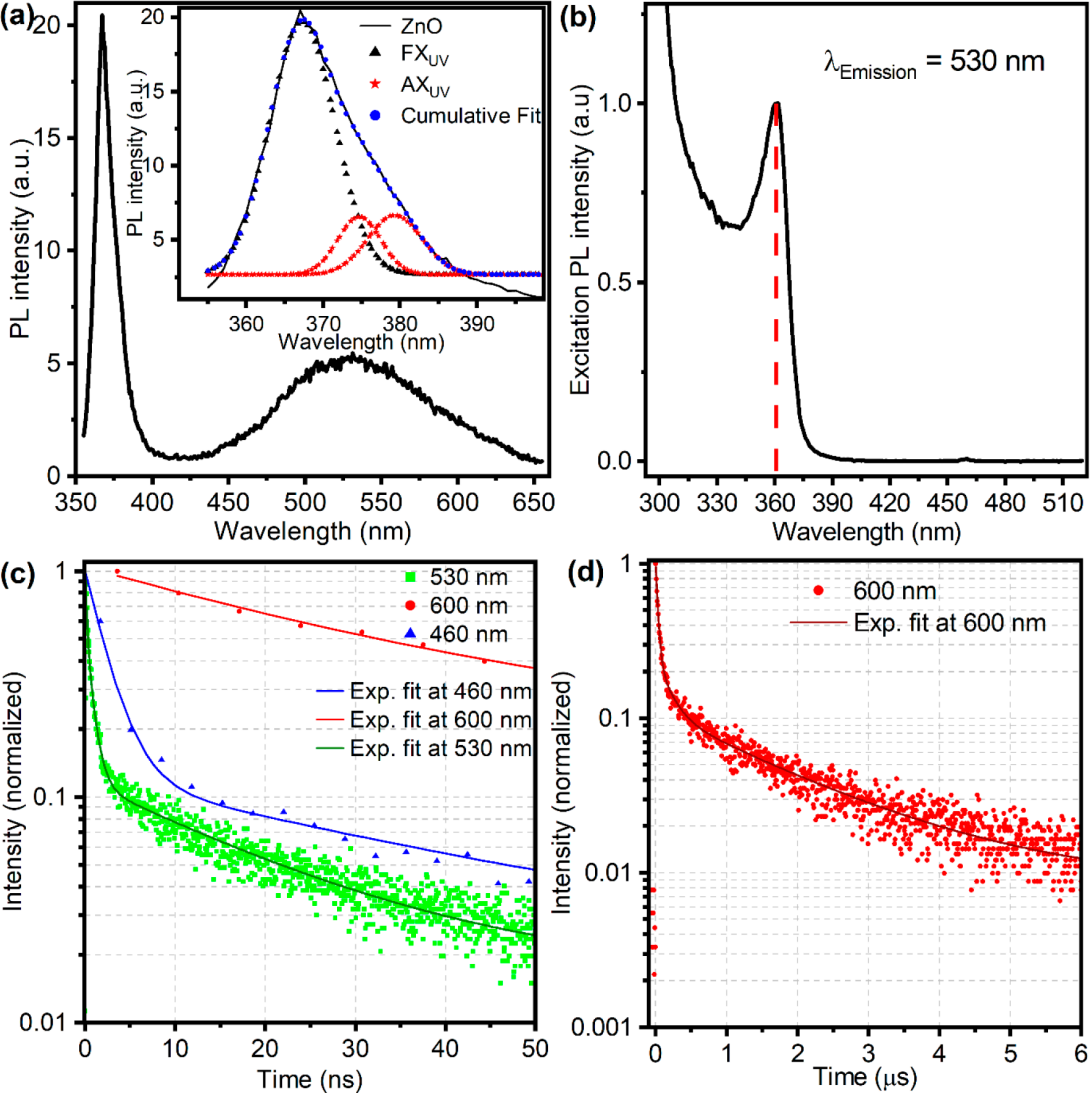
Room-temperature photoluminescence and excitation properties of ZnO QDs in ethanol. (a) Photoluminescence spectrum with excitation at 350 nm; the inset details the decomposition of the broad UV emission peak. (b) Excitation spectrum monitored at an emission wavelength of 530 nm. Dashed line indicates the sharp excitonic absorption. Time-resolved PL traces recorded at (c) 460, 530, and 600 nm over a 50 ns time scale and (d) 600 nm over a 6 μs time scale, illustrating the emission kinetics.

**Scheme 2:**
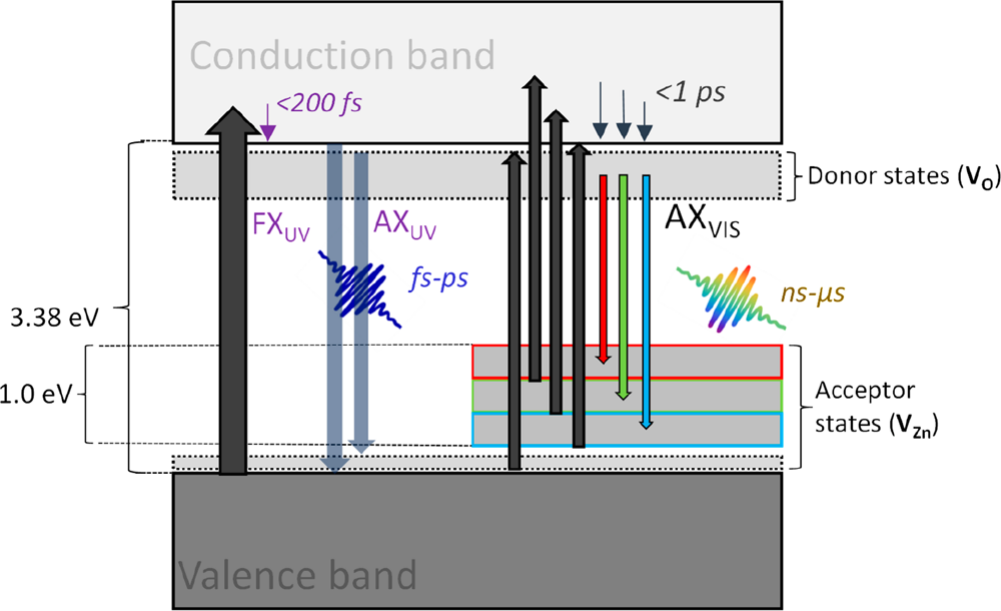
Overview of Carrier Dynamics through Valence Band, Conduction Band and Defect States, with Time Frames Excited carrier relaxation transitions by free-excitons (FX_UV_), acceptor defect-bounded excitons in UV region (AX_UV_), and acceptor defect-bounded excitons in visible region (AX_VIS_) are depicted.

In the photoluminescence excitation (PLE) experiment, we focused on the maximum peak of the broad visible emission band, located at around 530 nm. Figure 3b shows the PLE spectrum of ZnO QDs as a function of the excitation wavelength while specifically monitoring the light emitted at 530 nm. In the PLE spectrum (Figure 3b), the peak observed at approximately 362 nm corresponds to the excitation wavelength that efficiently leads to visible emission at 530 nm, suggesting a significant role of excitonic processes.^49^ These excitonic processes likely involve recombination events from the conduction band minimum to acceptor states, which are then manifested as visible emission, termed as a part of AX_VIS_. In the subsequent section, we delve deeper into the specifics of FX_UV_, AX_UV_, and AX_VIS_, including their concentration and nature as well as their influence on the p-type character of ZnO. This discussion will be supported by the results of our time-resolved spectroscopy analysis.

The nature of exciton relaxation can be more effectively explored through time-resolved experiments given the unique lifetimes of excitons in the conduction band and midgap states. Figure 3c shows kinetic traces obtained by time-correlated single-photon counting (TCSPC) experiments. We measured TCSPS signals at three selected wavelengths (460, 530, and 600 nm) spanning the broad visible band. The kinetic traces can be modeled well with three exponential decay constants, with fit parameters provided in Table 1. The commonly observed green emission at 530 nm demonstrates the shortest decay lifetimes (t1_green_ = 160 ps, t2_green_ = 775 ps, t3_green_ = 19.9 ns), in comparison to the lifetimes at 460 nm (t1_blue_ = 2.3 ns, t2_blue_ = 39.5 ns, t3_blue_ ≈ 1.1 μs) and 600 nm (t1_red_ = 30 ns, t2_red_ = 185 ns, t3_red_ ≈ 1.83 μs). While energetically the green emission sits between blue and red emissions in the spectrum, the characteristics of these emissions are not solely dictated by their energy levels. This observation of shorter lifetimes for green emission may stem from distinct recombination mechanisms, independent of the emission’s spectral location. It suggests that specific defect states, possibly more prevalent or efficient in facilitating nonradiative recombination, are involved.^50^ The long-lived visible emission line was assigned to V_Zn_ acceptor states which are interpreted as the main source of p-type ZnO^8,16,18,51^ and dominant compensation center in n-type ZnO.^8,20,52,53^ To shed light on the nature of the emission via charge carrier trapping events near the conduction band minimum, which occur at picosecond scales,^27^ we performed ultrafast transient absorption spectroscopy (TAS) and fluorescence up-conversion spectroscopy (FLUPS) measurements.

**Table 1:** Decay Constants of Transient Absorption (TA), Fluorescence Up-Conversion Spectroscopy (FLUPS), and PL Emission Kinetics Taken by Time-Correlated Single-Photon Counting Spectroscopy (TCSPC)

	362 nm (TA)	377 nm (FLUPS)	460 nm (TCSPC)	530 nm (TCSPC)	600 nm (TCSPC)
A1	8.5 × 10^–5^ ± 2.2 × 10^–5^	0.54 ± –	0.87 ± 0.002	0.46 ± 0.011	0.78 ± 0.0062
t1	0.6 ± 0.3 ps	0.66 ± 0.07 ps	2.31 ± 0.01 ns	0.16 ± 0.01 ns	30.2 ± 0.5 ns
A2	3 × 10^–4^ ± 3.4 × 10^–5^	0.59 ± –	0.106 ± 0.001	0.48 ± 0.01	0.15 ± 0.01
t2	24.7 ± 5.4 ps	17.4 ± 3.2 ps	39.5 ± 0.7 ns	0.78 ± 0.02 ns	185 ± 8 ns
A3	1.5 × 10^–3^ ± 1.7 × 10^–3^		0.015 ± 2.18 × 10^–4^	0.101 ± 6.84 × 10^–4^	0.102 ± 0.001
t3	1330 ± 1874		1096 ± 25 ns	19.9 ± 0.2 ns	1835 ± 24 ns

*Ultrafast Optical Spectroscopy Measurements*. TAS is based on a pump–probe technique where the material is excited by a pump beam and probed by a relatively weak broadband beam; the difference of the probe beam when the system is pumped and unpumped gives us the Δ*A* spectrum: Δ*A*_probe_ = *A*_probe(pumped)_ – *A*_probe(unpumped)_. Transient absorption spectra of ZnO QDs, upon excitation at 356 nm, are shown in Figure 4a. A negative ground-state bleach (GSB) peak appears at around 362 nm and indicates a decrease in ground-state absorption. GSB peak minimum aligns with the excitonic peak in the PLE spectra depicted in Figure 3b. Hence, the GSB dynamics also relates with midgap defect states, AX_VIS_.^22,49^ The kinetic trace at this wavelength shown in the inset of Figure 4a has been fit by a triexponential decay; the fit parameters are listed in Table 1. We find exponential decay constants of t1_TAS_ = 0.6 ± 0.4 ps, t2_TAS_ = 25 ± 5 ps, and t3_TAS_ > 1 ns. While t3_TAS_ indicates the time of GSB recovery through the carriers from long-lived defect states, t2_TAS_ indicates most likely the relaxation from conduction band to the defect states (trapping) and t1_TAS_ reveals the fast GSB recovery through exciton recombination in the UV domain as explained below.

**Figure 4:**
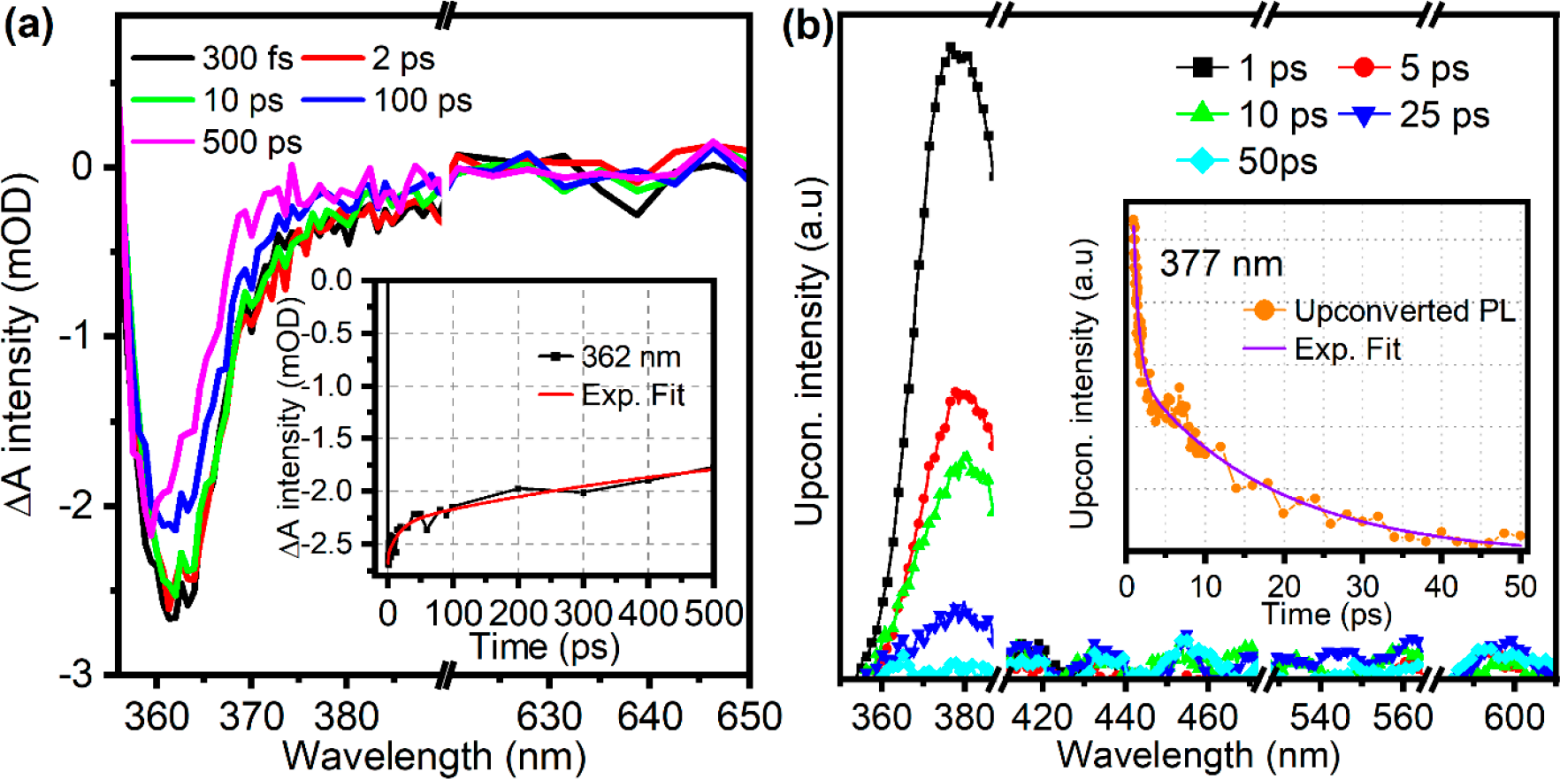
Time-resolved optical characterization of ZnO quantum dots. (a) Transient absorption spectroscopy (TAS) following excitation with a 356 nm pump beam, detailing the spectral evolution at various delay times with the inset capturing the decay kinetics at 362 nm. (b) Fluorescence up-conversion spectroscopy (FLUPS) initiated by a 350 nm excitation source, demonstrating the emission dynamics over time; the inset illustrates the decay profile at 377 nm, showcasing the temporal resolution of the upconverted photoluminescence.

Rapid GSB state refilling is primarily attributed to excitonic UV emission, as evidenced by FLUPS measurements with 350 nm excitation. The FLUPS spectrum (Figure 4b) displays decay of the FX_UV_ and AX_UV_ emission within 50 ps. A biexponential decay function fits this kinetic trace, with time constants t1_FLUPS_ = 0.7 ps and t2_FLUPS_ = 17 ps (Table 1), in accordance with t1_TAS_ and t2_TAS_. We suggest these reflect the FX_UV_ and AX_UV_ dynamics, respectively, as subsequent relaxation occurs after free carrier capture in defect states.^22,47^

*Discussion*. The primary significance of this research lies in bridging the knowledge gap regarding ZnO quantum dots that exhibit enhanced p-type conductivity due to the compensation of native defects synthesized using a straightforward and effective optimized sol–gel method. We employed time-resolved X-ray spectroscopy^36,54^ alongside optical time-resolved spectroscopy^22,27,28,50,55^ to unravel the complex carrier relaxation processes occurring within the midgap states of ZnO. These methods have substantially deepened our insights into the dynamics of photoexcited carriers in ZnO. Still, while ZnO is typically regarded as an n-type semiconductor due to V_O_’s, research on relaxation mechanism considering V_Zn_’s remains scarce, particularly for quantum dots, highlighting the novelty of our approach.

The sol–gel method is commonly used for the synthesis of ZnO nanoparticles, but it can result in residual materials that affect properties such as size, uniformity, and optical absorption and emission characteristics.^25,29,35^ The presence of byproducts in ZnO and the necessity for purification have been well-documented using various analytical methods.^30−32^ We have leveraged the sensitivity of Zn K-edge by XANES, which is element-specific and sensitive to local structure, to the coordination environment of Zn to better understand the chemical reactions during synthesis and the subsequent growth of ZnO.^38^ For example, during synthesis, the formation of soluble Zn(OH)_4_^2–^ can remain in the final colloidal mixture.

We suggested this by analyzing the Zn K-edge in Supernatant-II, which is expected to contain Zn(OH)_4_^2–^ in addition to the precursor molecules and by conducting a comparative assessment with the precursor. Further comparisons were made between the spectra of Supernatant-II and those of the final ZnO QDs. In Zn(OH)_4_^2–^, zinc is surrounded by hydroxide ions, which are not as electronegative as the oxygen in ZnO. This means the 1s electrons in zinc are more tightly bound and require more energy to excite to the 4p states, causing a shift in the Zn K-edge to higher energies compared to ZnO. This shift is also due to the more ionic Zn–OH bonds in the hydroxide complex, as opposed to the covalent Zn–O bonds in ZnO, affecting the electron distribution and the energy levels involved in the electron transitions. XANES analysis corroborates our hypothesis regarding the elimination of excess unreacted or intermediate reaction species, which are formed during the sol–gel method and removed by subsequent processes: In comparing the products of Supernatant-I (Scheme 1a) with the final ZnO QD (Scheme 1d), we can infer that a more efficient decomposition of Zn(OH)_4_^2^^–^ results in reduced residual zinc in the form of Zn(OH)_4_^2^^–^. This potentially allows for a greater level of oxygen incorporation into the ZnO structure. Moreover, the absence of pre-edge features in the Zn K-edge spectrum of ZnO QD, as shown by the green line in Figure 1a, implies insignificant amount of oxygen vacancies,^37^ a conclusion supported by EDX (Figure 2b) results consistent with an oxygen-rich nature of the chemical composition.

The EPR data in Figure 2a exhibit a low signal-to-noise ratio, which might be because EPR is inherently sensitive to only specific types of paramagnetic defects such as V_o_^+^ and V_Zn_^–^. EPR does not detect neutral (V_O_^0^ and V_Zn_^0^) or doubly charged (V_O_^2–^ and V_Zn_^2–^) defects. Previous EPR studies on ZnO have linked signals with *g*-values of approximately 2.00 and 1.96 to these paramagnetic defects.^56^ However, size-dependent EPR studies suggest that the *g* ≈ 1.96 signal, often associated with V_Zn_^–^, becomes less prominent in quantum dots.^44^ Theoretical work indicates that surface vacancies are likely positively charged V_o_, causing displacement, while core expansions are due to negatively charged V_Zn_, which leads to an outward shift of neighboring oxygen atoms.^20,57^ Although we cannot entirely rule out the presence of donor defects such as V_O_^0^ and V_O_^2–^ within the core,^36^ the EDX data suggest that our QDs contain a smaller proportion of oxygen vacancies relative to zinc vacancies.

Quantum confinement increases the exciton binding energy in ZnO QDs, leading to discrete energy levels and enhanced radiative recombination, which sharpen the UV emission peak with a decreasing particle size (Figure 3a). Smaller nanoparticles, due to their larger surface-to-volume ratio, exhibit fewer structural defects, minimizing nonradiative recombination and further refining the UV peak. The pronounced UV emission peak observed in ZnO at both nano and bulk scales typically manifests at low temperatures, as a result of diminished electron–lattice interactions and the nonlinear temperature-dependent behavior of the thermal expansion coefficient, as described by Varshni.^58^ Fonoberov et al. established that UV emissions in ZnO PL in QDs is mainly attributed to acceptor-bound excitons across all temperatures, in contrast to larger ZnO structures where different types of excitons dominate depending on the temperature.^23^ This observation suggests that a variety of recombination mechanisms exist between ZnO QDs and their larger counterparts. Our analysis correlates the excitonic peaks, FX_UV_ and AX_UV_, with these observations, as shown in Figure 3a and Scheme 2.^18,23^ Moreover, the pronounced peak at 362 nm in the PLE spectrum, when monitoring emission at 530 nm (Figure 3b), implies that many photoexcited electrons are relaxing via transitions that involve defect-bound states, underscoring the significant role of defect-related excitonic processes in the emission mechanism.^49^

The broad visible emission in the PL spectrum (Figure 3a), so-called green emission, has been debated due to its sensitivity to the synthesis procedure.^2,23,50^ Understanding the origin of the green emission and defect-related phenomena necessitates knowledge of size and chemical composition knowledge. A significant increase in the green emission band was observed with an increase in the surface-to-volume ratio from bulk to nano size.^2^ Before visible emission, the photoexcited electrons are expected to relax to the conduction band edge in the hot-carrier regime (<200 fs) and be trapped at shallow donor states in the nonthermal regime (<2 ps).^59^ In many ZnO systems, V_O_ is often associated with green emission, although the exact mechanism of green emission through shallow and deep defects is an open question.^2,20^ Due to the core–shell structure and shorter lifetime of green emission compared to red and blue emission (Figure 3c), we can assign the short lifetimes of green emission (t1_green_ = 160 ps, t2_green_ = 775 ps) to shallow (surface) V_O_ donor defect states as an initial state of green emission. Surface defects often have shorter lifetimes due to the increased availability of nonradiative recombination paths.^59^ Conversely, emissions associated with deeper or core-related defects, for instance V_Zn_, might exhibit longer lifetimes because they are more shielded from nonradiative surface processes. Hence, much longer lifetimes (t1_blue_ = 2.3 ns, t2_blue_ = 39.5 ns, t3_blue_ ≈ 1.1 μs, t1_red_ = 30 ns, t2_red_ = 185 ns, t3_red_ ≈ 1.83 μs, and t3_green_ = 19.9 ns) can be attributed to core V_Zn_ acceptor states as the final states of those emissions. Such long-lived carrier lifetimes have only been observed under substantial acceptor defect concentration close to the valence band.^22^

Previous studies have documented exciton lifetimes in bulk ZnO as 322 ps.^60^ In ZnO thin films, radiative decay of free excitons typically occurs within a few nanoseconds, whereas defect-bound excitons have been shown to emit for durations extending to 50 ns.^22^ In ZnO QDs in suspension, excitonic emission was observed to last less than 50 ps,^61^ aligning with our observations of UV exciton emission persisting for 50 ps (Figure 4b). Specifically, our analysis identifies two rapid decay constants, t1_FLUPS_ = 0.7 ps and t2_FLUPS_ = 17 ps (Table 1), which we attributed to FX_UV_ and AX_UV_, detailed in Scheme 2. These constants are consistent with the fast decay constants obtained from transient absorption spectroscopy (t1_TAS_ = 0.6 ± 0.4 ps and t2_TAS_ = 25 ± 5 ps), corroborating the assignment of these decay pathways to transitions between conduction band and valence band states, as well as to acceptor-bound excitons.^47,49^ In reality, we anticipate a more complex relaxation mechanism than what was presented in Scheme 2. This complexity arises because defects with varying charge states possess distinct energy levels, which are further modified following photoexcitation and the subsequent relaxation of carriers through these defect states over different time scales. Time-resolved x-ray spectroscopy, supplemented by thorough ab initio calculations as referenced in refs (36) and (54), can clarify this process.

The demand for compensating inherent donor defects for n-type ZnO-based heterojunctions or achieving purely p-type ZnO remains. The former is crucial for a range of biomedical applications, including optical neural interfaces,^6^ as well as photovoltaic^4^ and photocatalytic^5^ energy-harvesting technologies. The latter is desired for LEDs,^10^ dye-sensitized solar cells,^12^ gas sensing devices,^13^ and ferromagnetic semiconductors.^16,62−64^ Although intrinsic p-type ZnO faces challenges such as low hole mobility^65^ and the compensation of native n-type defects, such as oxygen vacancies,^66^ these issues are predominantly associated with Zn-rich ZnO. In contrast, the realization of p-type conductivity in ZnO under oxygen-rich conditions has been substantiated through both experimental^15,17,18^ and theoretical studies.^20,21,65,66^ We enhanced the sol–gel synthesis of ZnO QDs to remove intermediate reaction molecules and byproducts, fostering stronger p-type properties, primarily due to zinc vacancies in oxygen-rich conditions. Our investigation covers a comprehensive analysis of carrier recombination within the midgap states, extending from femtoseconds to microseconds, which supports the compensation of n-type defects in ZnO QDs.

In conclusion, our enhanced synthesis method produces uniformly distributed, oxygen-rich ZnO quantum dots, revealing pronounced UV emission at room temperature and suggesting enhanced p-type conductivity by compensating for inherent donor defects. This research is especially important considering the limited studies on carrier relaxation in ZnO quantum dots that focus on zinc vacancies as opposed to the more commonly explored oxygen vacancies. Our comprehensive analysis of carrier recombination dynamics spans femtoseconds to microseconds, shedding light on the fundamental defect-related mechanisms within the material. We conclude that the long-lived carriers, emitting visible light, are primarily linked to acceptor defect states, a finding that adds a unique perspective to our understanding of ZnO quantum dots.

## Supporting Information Available

Synthesis of the material, characterization of the material (SEM, XRD, EPR, and EDX), core-level spectroscopy (XAS) of the material, steady-state optical spectroscopy (PL, PLE) and time-resolved optical spectroscopy (TCSPC, FLUPS, TAS) of the material, and fitting procedures for time-resolved spectroscopy measurements (PDF)
Transparent Peer Review report available (PDF)
