# Supplementary material for: Tailoring p-Type Behavior in ZnO Quantum Dots through Enhanced Sol–Gel Synthesis: Mechanistic Insights into Zinc Vacancies
Source: J Phys Chem Lett. 2024 Feb 7;15(6):1755–64. doi: 10.1021/acs.jpclett.3c03519 (PMC10875662; doi:10.1021/acs.jpclett.3c03519)
Supplement: Supplementary file 1 — jz3c03519_si_001.pdf [file jz3c03519_si_001.pdf]

Supporting information

Tailoring p-type Behavior in ZnO Quantum Dots through Enhanced Sol-Gel Synthesis: Mechanistic Insights into Zinc Vacancies

Abdullah Kahraman[1]†#, Etienne Socie[2], Maryam Nazari[3], Dimitrios Kazazis[1], Merve Buldu-Akturk[4], Victoria Kabanova[1], Elisa Biasin[5], Grigory Smolentsev[1], Daniel Grolimund[1], Emre Erdem[4], Jacques E. Moser[2], Andrea Cannizzo[3], Camila Bacellar[1]* and Christopher Milne[6]**

1. *Paul Scherrer Institut, CH-5232 Villigen PSI, Switzerland*
2. *École polytechnique fédérale de Lausanne (EPFL), Rte Cantonale, 1015 Lausanne, Switzerland*
3. *Institute of Applied Physics, University of Bern, Sidlerstrasse 5, 3012 Bern, Switzerland*
4. *Faculty of Engineering and Natural Sciences, Sabanci University, Tuzla 34956, Istanbul, Turkey.*
5. *Physical Sciences Division, Pacific Northwest National Laboratory, Richland, WA 99352, USA.*
6. *European XFEL GmbH, 22869 Schenefeld, Germany.*

Corresponding Author

*Abdullah Kahraman, *Camila Bacellar, *Christopher Milne

†Physical Sciences Division, Pacific Northwest National Laboratory, Richland, WA 99352, USA.

# Stanford PULSE Institute, SLAC National Accelerator Laboratory, Stanford University, Menlo Park, CA 94025, USA.

Table of Contents

Synthesis……………………………………………………………………………….. S3

Experimental Methods ..…………………………………………………………………...S3

Fitting procedures of optical time-resolved spectroscopy measurements……………………S6

References………………………………………………………………………………S7

Synthesis

We prepared a colloidal solution of ZnO by modifying the sol-gel method recipe as described by Ullah et al. [1]. The precursor was prepared using zinc acetate dihydrate $Zn(CH_3-COO)_2.2H_2O$ and absolute ethanol, both of 99.9% purity from Sigma-Aldrich. The sol-gel formation process can be divided into four steps: hydrolysis, condensation and polymerization (nucleation) of monomers to form particles, particle growth, and aging [2]. In a typical synthesis, we dissolved 2 mmol of zinc acetate dihydrate in 20 ml of ethanol and heated it to approximately 60 °C with vigorous stirring until it completely dissolved. This precursor solution was then complexed with 20 ml of pure ethanol, and the mixture was heated at around 85 °C for 30 minutes. After cooling to room temperature, the solution was mixed with 20 ml of 2 mmol sodium hydroxide for hydrolysis. The solution was then kept at around 67 °C in a water bath for two hours and left to stand at room temperature for approximately 18 hours. We used centrifugation for 20 minutes at 4000 rpm. The supernatant part involves ZnO along with other unreacted/intermediate hydroxy groups mentioned in the manuscript while the precipitated part involves larger particles. We did further purification and processes which can be found in the main text.

Experimental methods

We obtained scanning electron microscopy (SEM) images using a Hitachi Regulus 8230 ultra-high-resolution SEM operated at an accelerating voltage of 5kV, immediately after ethanol evaporation on a silicon wafer. The EDX spectrum was taken with an Oxford windowless detector (Ultim Extreme), ideal for EDX analysis at low accelerating voltages. The detector was used in the same SEM and the EDX spectra were acquired at an accelerating voltage of

5keV. The average count rate during acquisition was 7944 cps. The total number of counts collected for the spectrum was 534144 counts. To properly plot the cps/eV versus energy we divided the counts by the total live time (67.238090515 s) and by the energy resolution (energy per channel): 0.01 eV. The atomic composition was calculated using Oxford's Aztec software, excluding carbon which was likely due to sample contamination.

We carried out powder X-ray diffraction (XRD) measurements on a Bruker D8 Advance type diffractometer using a copper photocathode ($\lambda 1$ = 1.54060 Å, $\lambda 2$ = 1.54439 Å, $I\lambda 1/I\lambda 2$ = 0.5). Before resuspension, the precipitated part was dried at 80 °C for 1 hour for XRD measurement. We conducted the Zn Kedge XAS measurements at the microXAS beamline of the Swiss Light Source synchrotron. The colloidal dispersion flows through the cell with 800 μm jet thickness by using a peristaltic pump. 50 ml reservoir has been used with magnetic stirrer and changed time to time with fresh samples. X-ray spot size was approximately 30μm. A single-element silicon drift detector (Ketek AXAS-SDD10-138500, 10 mm2) with an energy resolution of ~150 eV ($\Delta E/E \sim 1.5\%$) was used for fluorescence detection.

We used a Bruker EMX-Nano spectrometer for electron paramagnetic resonance (EPR) measurements, with an integrated referencing for g-factor calculation and integrated spin counting units. The microwave frequency of the resonator was in X-band (9.64 GHz) region and all spectra were measured at room temperature with 1 G modulation amplitude, 2 mW microwave power, and 50 scans. Each scan has a sweep time and time constant of 120 s and 81.92 ms, respectively. Samples were inserted into spin-free 25 cm long quartz tubes (Qsil, Germany). The calculation of g factor is given by resonance condition hf=gbB where h is planck constant, f is microwave frequency (here, 9.64 GHz), b is the Bohr magneton and B is the external applied magnetic field. Unlike NMR, in EPR we scan the magnetic field and keep

the frequency fixed. Therefore, by reading out the magnetic field at the middle of first derivative EPR signal we obtain the value of B. The rest is simple calculation of given equation. Nowadays, all the EPR related software compute the g-factors automatically.

We carried out the powder XRD measurements on a Bruker D8 Advance type diffractometer using a copper photocathode (λ1 = 1.54060 Å, λ2 = 1.54439 Å, Iλ1/Iλ2 = 0.5). The samples in ethanol were dried at room temperature. We calculated particle size for (002) plane as 4.16 nm through Debye Scherrer equation [3]:

$$D = \frac{K\lambda}{\beta cos(\theta)} \tag{1}$$

where D is the crystal size, K is the shape factor (0.9), λis the x-ray wavelength (0.154 nm), β is the full width half maximum of the peak in radians (~0.3054 radians), θ is the Bragg angle for the (002) plane (17.5 degrees).

Steady-state PL and time-correlated single photon counting (TCSPC) measurements were performed on a Horiba JobinYvon Fluorolog-3 instrument. The excitation source for TCSPC was a nano LED N-390 system with a repetition rate of 1 MHz, a pulse duration of 1.3 ns, and an excitation wavelength λ_{exc}= 350 nm. The PL detection was performed at 90°relative to the excitation source with a photomultiplier tube, with other details available in the reference[4].

A broadband fluorescence up-conversion setup (FLUPS,LIOP-TEC) was used for ultrafast time-resolved PL measurements. The pump pulse (λ_{exc}= 350 nm) was generated by optical parametric amplifer followed by frequency doubling the output of a chirped pulse-amplified(CPA) Ti:sapphire laser (Libra-HE USP, Coherent) and focused onto a 150μm spot at the sample position. The gate pulse (λ_G= 1300 nm) was obtained from a white light-seeded

optical parametric amplifier (OPerA-Solo, Coherent). The upconverted fluorescence was generated by type II sum-frequency generation in a 100µm-thick BBO crystal (EKSMA Optics). The signal was dispersed in wavelength utilizing UV-grating and directed to a CCD camera (Newton 920, Andor). The time correction for the impulse response function (IRF) was calculated to be 250 fs using the cross-correlation between the pump and the probe.

We performed transient Absorption spectroscopy experiments upon excitation at 356 nm, focusing into 60 µm diameter ($1/e^2$) with energy 63 uJ/cm^2 with other details available in the reference [5]. The pump pulse (λ_{exc}= 356 nm) was generated by optical parametric amplifer (Topas White by Light conversion) followed by frequency doubling the output of a 5kHz chirped pulse-amplified(CPA) Ti:sapphire laser (Legend Elite by Coherent).

Fitting procedures of optical time-resolved spectroscopy measurements

Fitting equation used for FLUPS kinetic measurements probed at 377 nm:

y = y0 + A1*exp(-(x-x0)/t1) + A2*exp(-(x-x0)/t2)

Fitting equation for TAS kinetic measurements probed at 362 nm and tr-PL (TCSPC) kinetic measurements probed at 460 nm, 530 nm and 600 nm:

y = A1*exp(-x/t1) + A2*exp(-x/t2) + A3*exp(-x/t3) + y0

References

There are no sources in the current document.

(1) Ullah, R.; Dutta, J. Photocatalytic degradation of organic dyes with manganese-doped zno nanoparticles. *J Hazard Mater* **2008**, *156* (1-3), 194-200. DOI: 10.1016/j.jhazmat.2007.12.033.

(2) Alias, S. S.; Ismail, A. B.; Mohamad, A. A. Effect of ph on zno nanoparticle properties synthesized by sol-gel centrifugation. *J Alloy Compd* **2010**, *499* (2), 231-237. DOI: 10.1016/j.jallcom.2010.03.174.

(3) Holder, C. F.; Schaak, R. E. Tutorial on powder x-ray diffraction for characterizing nanoscale materials. *Acs Nano* **2019**, *13* (7), 7359-7365. DOI: 10.1021/acsnano.9b05157.

(4) Socie, E.; Vale, B. R. C.; Terpstra, A. T.; Schiavon, M. A.; Moser, J. E. Resonant band-edge emissive states in strongly confined cspbbr
perovskite nanoplatelets. *J Phys Chem C* **2021**, *125* (26), 14317-14325. DOI: 10.1021/acs.jpcc.1c01353.

(5) Pashaki, M. N. H.; Choi, T. K.; Rohwer, E. J.; Feurer, T.; Duhme-Klair, A. K.; Gawelda, W.; Cannizzo, A. Unveiling the origin of photo-induced enhancement of oxidation catalysis at mo(vi) centres of ru(ii)-mo(vi) dyads†. *Chem Commun* **2021**, *57* (34), 4142-4145. DOI: 10.1039/d1cc00750e.
