# Supplementary material for: Tailoring p-Type Behavior in ZnO Quantum Dots through Enhanced Sol–Gel Synthesis: Mechanistic Insights into Zinc Vacancies
Source: J Phys Chem Lett. 2024 Feb 7;15(6):1755–64. doi: 10.1021/acs.jpclett.3c03519 (PMC10875662; doi:10.1021/acs.jpclett.3c03519)
Supplement: Supplementary file 2 — jz3c03519_si_002.pdf [file jz3c03519_si_002.pdf]

jz-2023-03519p.R1

Name: Peer Review Information for "Tailoring p-type Behavior in ZnO Quantum Dots through Enhanced Sol-Gel Synthesis: Mechanistic Insights into Zinc Vacancies"

First Round of Reviewer Comments

Reviewer: 1

Comments to the Author

The research presented in this article is highly commendable as it enhances our understanding of the synthesis and control of p-type ZnO properties, which is crucial for improving optoelectronic and spintronic devices. The authors employ an innovative approach using an enhanced sol-gel process to produce colloidal ZnO quantum dots. This approach not only addresses the challenge of controlling intrinsic midgap states but also offers a potential methodology for achieving p-type conductivity by compensating for common n-type defects.

While the study provides valuable insights, several questions arise that could further clarify the research's scope and applicability. Here are the specific questions:

1. Can the authors provide more details on the mechanism by which ethanol evaporation contributes to the enhanced dispersion and stabilization of ZnO nanoparticles? Additionally, is there quantitative evidence available to support this claim?

2. The article mentions that maintaining a temperature of 68°C helps reduce sedimentation. What criteria were used to select this specific temperature, and is there a comparative study available that demonstrates the superiority of 68°C over other temperatures in mitigating sedimentation?

3. Regarding the assertion of a thin passivation layer or shell structure forming on ZnO, are there Transmission Electron Microscopy (TEM) images or other empirical evidence available to confirm the existence of such a structure?

4. Can a higher resolution Scanning Electron Microscopy (SEM) image of Figure 1(b) be provided for better clarity? Additionally, for part (c), can X-ray diffraction (XRD) patterns be supplemented with standard reference spectra to confirm adherence to the standard sphalerite crystal structure?

5. In the section discussing the assessment of defects through Electron Paramagnetic Resonance (EPR) and Energy Dispersive X-ray (EDX) spectroscopy, can the authors provide a comprehensive description of the methodology used to compute the g-factor values? Can the parameters and equations employed in the calculation be disclosed?

6. The article mentions that transitioning from bulk to nanoscale dimensions leads to a reduction in core signal intensity and an amplification of surface defect signals. Is it possible to quantify this variation in intensity and provide a graphical representation of these changes?

7. Can the authors provide further clarification on how quantum confinement effects influence the sharpness of the 367 nm peak observed in ZnO? Additionally, in a quantitative sense, how does this effect compare with the properties of bulk and nanocrystalline ZnO at the specified temperatures?

Reviewer: 2

Comments to the Author

The work has been revised partly. Major revisions are still needed.

For claiming these particles as QDs, high resolution TEM images are necessary. SEM imaging is not enough.

There is no clear evidence of the defect states from the XANES plots in Fig. 1a. Also, please check that any EDX analyzer can detect elements from and above the atomic number 11. Oxygen has an atomic number of 8. I suggest the authors to carry out Rietveld refinement of the X-ray diffraction pattern (measured in a slow scan) to estimate any cation / anion deficiency.

Author's Response to Peer Review Comments:

Abdullah Kahraman
3943 El Camino Real 7
Palo Alto, 94306, CA
abdullah.kahraman@pnnl.gov, kahraman@slac.stanford.edu

01/14/2024

Dear Prof. Editor,

I am writing this letter in reply to the editorial request for the revision regarding manuscript number jz-2023-03519p, entitled 'Tailoring p-type Behavior in ZnO Quantum Dots through Enhanced Sol-Gel Synthesis: Mechanistic Insights into Zinc Vacancies'. In the following sections, I address the reviewer's comments and questions responding in red italic text while I highlighted in yellow and writing red, italic and bold for the changes we made after valuable comments and questions. I am enclosing an updated version of the manuscript.

Reviewer-1's comments:

The research presented in this article is highly commendable as it enhances our understanding of the synthesis and control of p-type ZnO properties, which is crucial for improving optoelectronic and spintronic devices. The authors employ an innovative approach using an enhanced sol-gel process to produce colloidal ZnO quantum dots. This approach not only addresses the challenge of controlling intrinsic midgap states but also offers a potential methodology for achieving p-type conductivity by compensating for common n-type defects.

Answer: We are appreciative of such supportive feedback, we thank the reviewer for this comment.

While the study provides valuable insights, several questions arise that could further clarify the research's scope and applicability. Here are the specific questions:

1. Can the authors provide more details on the mechanism by which ethanol evaporation contributes to the enhanced dispersion and stabilization of ZnO nanoparticles? Additionally, is there quantitative evidence available to support this claim?

Answer: *By evaporating ethanol, we increased the ZnO concentration. This step caused increased turbidity, an indicator of enhanced number of suspended particles. Enhanced dispersion or turbidity was very clear to naked eye; therefore, we did not further quantify the turbidity. Although this could have been quantified by zeta-potential measurement we did not have this opportunity. We show our modified version of this section after the second question because the modifications we made for these two questions overlap.*

2. The article mentions that maintaining a temperature of 68°C helps reduce sedimentation. What criteria were used to select this specific temperature, and is there a comparative study available that demonstrates the superiority of 68°C over other temperatures in mitigating sedimentation?

Answer: *Our criteria was two-fold to evaporate ethanol in a controlled way: First we wanted to limit the evaporation to 20% of the solution volume and second to keep the temperature similar to that of the hydrolysis step, which was 67 C as indicated in SI. The evaporation step could also have been performed at a lower temperature, but it would have taken longer. Higher temperatures made evaporation faster, but resulted in particles sticking to the inner wall of the sample bottle, making this less suitable. Temperature plays a critical role in sedimentation processes as shown in the ref. 34. This comparative study indicated that the increased suspension and subsequent decrease of the suspension as temperature increases. The optimum temperature for maximum suspension changes due to synthesis type and conditions for nanoparticle fabrication.*

We show here in bold how we modified the section to address the first two comments:

We initiated our modification process by heating (Scheme 1b), during which approximately 20% of the ethanol evaporated from uncapped bottles at a temperature of 68°C ***for two hours. This temperature helped us to evaporate ethanol in a controlled way.*** ~~*This step caused*~~ *Increased ZnO concentration improved turbidity* ***seen clearly by naked eye****, an indicator of enhanced suspendability [34].* ***This indicates a more uniform distribution of nanoparticles in the solution, reducing their tendency to settle quickly. This can happen when the particles are well-dispersed and stabilized in the solvent [34].*** ~~*This step enhanced the dispersion and stabilization of the nanoparticles, ensuring a more uniform distribution within the solution and minimizing sedimentation [34].*~~

3. Regarding the assertion of a thin passivation layer or shell structure forming on ZnO, are there Transmission Electron Microscopy (TEM) images or other empirical evidence available to confirm the existence of such a structure?

Answer: *We investigated the core-shell structure analysis of ZnO nanoparticles in our previous studies and referred to these at specific points. Though TEM was the method we originally wanted to utilize; we did not have opportunity to use TEM. In the manuscript we used language to indicate this core-shell structure as only being a possibility: "…This process could lead to the formation of a thin passivation layer or shell structure of ZnO, similar to what has been previously reported in the literature [35].", we did not make any further changes here.*

4. Can a higher resolution Scanning Electron Microscopy (SEM) image of Figure 1(b) be provided for better clarity? Additionally, for part (c), can X-ray diffraction (XRD) patterns be supplemented with standard reference spectra to confirm adherence to the standard sphalerite crystal structure?

Answer: *We appreciate the suggestion for higher resolution SEM imaging. While we did not have access to TEM, we utilized the Hitachi Regulus 8230 ultra-high-resolution SEM, which provided sufficient resolution to distinguish features in the nanometer range.*

ZnO is known to crystallize predominantly in the wurtzite structure, which is a hexagonal phase, rather than the sphalerite structure, which corresponds to cubic ZnS. The XRD pattern presented in our study shows peaks that are indexed to the hexagonal wurtzite phase of ZnO, with the characteristic peaks at 2θ values corresponding to the (100), (002), and (101) planes, among others, which are typical for wurtzite ZnO. The diffraction peaks at $2\vartheta \approx 31.7°$, 34.4°, 36.2°, 47.5°, 56.6°, 62.8°, and 67.9° can be assigned to the (100), (002), (101), (102), (110), (103), and (112) planes of the hexagonal wurtzite structure, respectively (JCPDS Card No. 36-1451). These assignments are consistent with the literature and standard reference patterns for wurtzite ZnO. Thus, our XRD data align with the expected

diffraction pattern for wurtzite ZnO, confirming the hexagonal phase of the quantum dots synthesized in our study. We believe that our characterization accurately reflects the crystal structure of our ZnO QDs, and we have presented the XRD data with the appropriate phase identification.

5. In the section discussing the assessment of defects through Electron Paramagnetic Resonance (EPR) and Energy Dispersive X-ray (EDX) spectroscopy, can the authors provide a comprehensive description of the methodology used to compute the g-factor values? Can the parameters and equations employed in the calculation be disclosed?

Answer: *Thank you for pointing out the description of the methodology of EPR and EDX analysis.*

For EPR, we described the measurement in detail in the supplementary information (SI) as:
We used a Bruker EMX-Nano spectrometer for electron paramagnetic resonance (EPR) measurements, with an integrated referencing for g-factor calculation and integrated spin counting units. The microwave frequency of the resonator was in X-band (9.64 GHz) region and all spectra were measured at room temperature with 1 G modulation amplitude, 2 mW microwave power, and 50 scans. Each scan has a sweep time and time constant of 120 s and 81.92 ms, respectively. Samples were inserted into spin-free 25 cm long quartz tubes (Qsil, Germany).

Now, we added the details of the calculation just as a continuation of the paragraph above:

The calculation of g factor is given by resonance condition hf=gbB where h is planck constant, f is microwave frequency (here, 9.64 GHz), b is the Bohr magneton and B is the external applied magnetic field. Unlike NMR, in EPR we scan the magnetic field and keep the frequency fixed. Therefore, by reading out the magnetic field at the middle of first derivative EPR signal we obtain the value of B. The rest is simple calculation of given equation. Nowadays, all the EPR related software compute the g-factors automatically.

For EDX, we believe that we explained the method in detail in SI as much as we can:
The detector was used in the same SEM and the EDX spectra were acquired at an accelerating voltage of 5keV. The average count rate during acquisition was 7944 cps. The total number of counts collected for the spectrum was 534144 counts. To properly plot the cps/eV versus energy we divided the counts by the total live time (67.238090515 s) and by the energy resolution (energy per channel): 0.01 eV. The atomic composition was calculated using Oxford's Aztec software, excluding carbon which was likely due to sample contamination.

6. The article mentions that transitioning from bulk to nanoscale dimensions leads to a reduction in core signal intensity and an amplification of surface defect signals. Is it possible to quantify this variation in intensity and provide a graphical representation of these changes?

Answer: *Thank you for highlighting the need to clarify the relationship between core-shell structures and defect signals. Our manuscript references our prior studies to support the expected EPR signal variation as ZnO transitions from bulk to nanoscale. We hope that the added clarifications in bold address your question:*

In nanoscale ZnO, distinct EPR signals with g-factor values around 1.96 and 2.00 are typically expected [2]. ***We previously showed that t~~T~~he*** *transition from bulk to nano-scale dimensions induces a decrease in the intensity of the g~1.96 signal which* ***~~may~~*** *even vanish**es** in QD size, coupled with an enhancement of the g~2.00 signal* ***[42-46]****. This phenomenon is best explained by a core-shell model [42, 45], where the g~1.96 signal is attributed to defects within the lattice core, while the g~2.00 signal originates from defects located at the surface. Shifting from bulk to nano-scale dimensions results in a diminished intensity of the core signal, accompanied by an increase in the surface defect signal [42-44, 46]. This is because, as ZnO transitions from bulk to nano form, the ratio of surface atoms to volume atoms increases.*

7. Can the authors provide further clarification on how quantum confinement effects influence the sharpness of the 367 nm peak observed in ZnO? Additionally, in a quantitative sense, how does this effect compare with the properties of bulk and nanocrystalline ZnO at the specified temperatures?

Answer: Your question provides an excellent opportunity to delve deeper into the nuances of quantum confinement and its implications on the optical properties of ZnO quantum dots (QDs). We appreciate once again because we believe that describing the quantum confinement effect in more detail will benefit the reader. There are multiple reasons of having sharper UV emission peak in QDs compared to nanoparticles and bulk that we can explain confidently. This question led us to dig into literature deeper and we found that Varshni discussed temperature - bandgap relation at low and high temperature by showing a mathematical relation. We added a sentence into the Results section (bold). We also changed the paragraph in the Discussion section significantly that we contrast as Old and New paragraph to highlight the changes below:

Results

The 367 nm peak is due to excitonic recombination and its sharpness is due to the significant quantum confinement effect displayed by ZnO QDs [23], a phenomenon that has been similarly observed in studies for bulk and nanocrystalline ZnO at only low temperatures - 100 K [22] and at 2 K [47] respectively: ***At higher temperatures electron -lattice interaction distorts the emission which is minimized in QDs due to well-confined (discrete) energy levels****.*

Discussion (Old)

As changes in size (bulk or nanoparticles or QDs), temperature variations also significantly influence the excitonic recombination processes in ZnO, including the FX_{UV}, AX_{UV}, and AX_{VIS} mechanisms (Scheme 2). In ZnO QDs, acceptor impurities predominantly control exciton recombination, whereas in ZnO nanoparticles, their influence is notable only at low temperatures (below 150 K). For bulk ZnO, these impurities do not significantly contribute to exciton recombination as observed in studies [23]. Furthermore, donor-type defects are not expected to lead to intense UV emission at room temperature, as shown in Figure 3a, due to a high likelihood of donor-bound exciton trapping and increased recombination rates [16, 18, 36, 59]. The pronounced peak at 362 nm in the PLE spectrum, when monitoring emission at 530 nm (Fig. 3b), implies that many photoexcited electrons are relaxing via transitions that involve defect-bound states.

Discussion (New)

Quantum confinement increases the exciton binding energy in ZnO QDs, leading to discrete energy levels and enhanced radiative recombination, which sharpens the UV emission peak with decreasing particle size (Fig. 3a).

Smaller nanoparticles, due to their larger surface-to-volume ratio, exhibit fewer structural defects, minimizing non-radiative recombination and further refining the UV peak. The pronounced UV emission peak observed in ZnO at both nano and bulk scales typically manifests at low temperatures, a result of diminished electron-lattice interactions and the non-linear temperature-dependent behavior of the thermal expansion coefficient, as described by Varshni [58]. Fonoberov et al. established that UV emissions in ZnO QDs are mainly due to acceptor-bound excitons across all temperatures, a contrast to the behavior in larger ZnO structures where different exciton types dominate depending on temperature [23]. This suggests varied recombination mechanisms between ZnO QDs and their larger counterparts . Our analysis aligns excitonic peaks FX_{UV} *and* AX_{UV} ***with these observations (Fig. 3a and Scheme 2) [18, 23]. Moreover, the pronounced peak at 362 nm in the PLE spectrum, when monitoring emission at 530 nm (Fig. 3b), implies that many photoexcited electrons are relaxing via transitions that involve defect-bound states, suggesting a significant role of excitonic processes [49].***

Reviewer-2's comments:

The work has been revised partly. Major revisions are still needed.

# For claiming these particles as QDs, high resolution TEM images are necessary. SEM imaging is not enough.

Answer: Thank you for your insightful comments regarding the characterization of the nanoparticles in our study. We understand and appreciate the importance of high-resolution TEM in providing detailed insights into the crystalline structure and precise size distribution of QDs. We strongly agree that TEM would help us to image QDs with better resolution especially having chance to image possible core-shell structure. However, for the scope and objectives of this project, we relied on scanning electron microscopy (SEM) for several reasons.

Resolution and Size Determination: Our SEM images clearly show particles with a uniform size of approximately 4.5 nm. While SEM typically offers lower resolution compared to TEM, modern SEM equipment is capable of achieving resolutions that are sufficient to resolve nanoparticles in the range of a few nanometers, as demonstrated in our study and other studies. We utilized Hitachi Regulus 8230 ultra-high-resolution SEM as we stated in SI along with other details of this measurement.

There are precedents in the scientific literature where SEM has been used for initial size characterization of nanoparticles, particularly when the particles are of a size that falls within the resolving power of the SEM used. Moreover, the resolution of our SEM images is very similar to TEM images of colloidal ZnO shown by Bahneman et al, referenced as 61 in the main text.

We supplemented our SEM analysis with other characterization techniques, such as broad XRD peaks to confirm the crystalline nature and estimation of particle size of the nanoparticles. Due to Debye-Scherrer formula, our QDs particle size was determined to be 4.16 nm. We have added these details into the new manuscript and SI.

# There is no clear evidence of the defect states from the XANES plots in Fig. 1a. Also, please check that any EDX

analyzer can detect elements from and above the atomic number 11. Oxygen has an atomic number of 8. I suggest the authors to carry out Rietveld refinement of the X-ray diffraction pattern (measured in a slow scan) to estimate any cation / anion deficiency.

Answer: *As we stated in the manuscript, the full d^{10} configuration of Zn^{2+} excludes a pre-edge feature in ZnO's spectrum unless oxygen vacancies modify the local zinc oxidation state and perturb the 3d orbitals. A pre-edge peak, due to the absence of oxygen can be expected below 9660 eV, as we referred to our previous study [37] in the text. In our current study, such pre-edge peak was notably absent in our spectra, suggesting a low concentration of oxygen vacancy (V_O) in our ZnO QDs.*

Older EDX analyzers were limited to detecting elements from with atomic numbers from 11 (Na) onwards. In this work we utilized a state-of-the-art Oxford Ultim Extreme windowless detector, which is optimized for operation at low keV and is suitable for light elements detection, down to Li. Therefore, detecting oxygen was not a problem for this detector.

We appreciate the idea of Rietveld refinement of the X-ray diffraction pattern to estimate any cation / anion deficiency. While Rietveld refinement is effective for understanding crystallographic structure, it might not be sensitive enough to accurately quantify the presence of point defects like zinc or oxygen vacancies of nanoparticles. These vacancies may only induce subtle changes in the diffraction pattern, especially in a nanomaterial where disorder and surface effects are pronounced. Besides, nanoparticles have broadened diffraction peaks, as in our case, due to size effects, which can complicate peak fitting during Rietveld refinement and reduce the precision of structural determination. Moreover, even if slight changes due to vacancies are detected in the diffraction pattern, attributing them unequivocally to specific types of vacancies (zinc or oxygen) can be challenging: Nanoparticles have a high surface-to-volume ratio, leading to increased surface contributions in XRD patterns. These surface effects can be challenging to account for in Rietveld refinement, potentially impacting the accuracy of structural determination based on defects where we propose zinc vacancies locates in the core and oxygen vacancies locates on the surface.

Thank you for valuable comments made by both reviewers.

Yours sincerely,

Dr. Abdullah Kahraman
Research Associate

Pacific Northwest National Laboratory (PNNL)
Physical Sciences Division - Chemical Physics Experimental
902 Battelle Blvd, Richland, WA 99354

Stanford PULSE Institute
SLAC National Accelerator Laboratory
2575 Sand Hill Road Menlo Park, California 94025

jz-2023-03519p.R2

Name: Peer Review Information for "Tailoring p-type Behavior in ZnO Quantum Dots through Enhanced Sol-Gel Synthesis: Mechanistic Insights into Zinc Vacancies"

Second Round of Reviewer Comments

Reviewer: 2

Comments to the Author

The authors have not devoted a minimal effort to address the comments on TEM or Rietveld analysis of X-ray diffraction patterns. Hence, I cannot recommend this work for JPC Letters.

Author's Response to Peer Review Comments:

Dear Prof. Editor,

I am very happy to hear your response. Thank you very much.

I did the non-scientific changes by adding SI summary to the manuscript as indicated in your email. I also fixed some typos and made small changes in a few lines for better clarification after reading the manuscript once again. I indicated the changes clearly in a new Cover Letter document (named as "Cover_letter_about_only_small_changes_for_clarification").

Best regards

Dear Prof. Editor,

Thank you for giving me the chance to add supplementary information (SI) and address minor details in the manuscript. As I mentioned in my previous response, I have thoroughly re-read the entire manuscript. I corrected some typos and made a few adjustments for better clarification. Additions are highlighted in bold, and deletions are indicated by using strikethrough on the words.

These excitonic processes likely involve recombination events from the conduction band minimum to acceptor states, which are then manifested as visible emission, referred to here **as a part of** AX_{VIS}.

A negative ground-state bleach (GSB) peak appears at around 362 nm and indicates a decrease of ground-state absorption. GSB peak minimum aligns with the excitonic peak in the PLE spectra depicted in Fig. 3~~a~~**b**.

PL in QDs ~~are~~ **is** ~~mainly~~ **attributed** ~~due~~ to acceptor-bound excitons across all temperatures, ~~a~~ **in** contrast to ~~the behavior in~~ larger ZnO structures where different ~~exciton~~ types **of excitons** dominate depending on temperature [23].
This **observation** suggests **that** varied recombination mechanisms **exist** between ZnO QDs and their larger counterparts.

Our analysis ~~aligns~~ **correlates the** excitonic peaks**,** FX_{UV} and AX_{UV}**,** with these observations ~~(~~ **as shown in** Fig. 3a and Scheme 2~~)~~ [18, 23].

Moreover, the pronounced peak at 362 nm in the PLE spectrum, when monitoring emission at 530 nm (Fig. 3b), implies that many photoexcited electrons are relaxing via transitions that involve defect-bound states, **underscores the** ~~suggesting a~~ significant role of **defect-related** excitonic processes **in the emission mechanism** [49].

Yours sincerely,

Dr. Abdullah Kahraman
Research Associate

Pacific Northwest National Laboratory (PNNL)
Physical Sciences Division - Chemical Physics Experimental
902 Battelle Blvd, Richland, WA 99354

Stanford PULSE Institute
SLAC National Accelerator Laboratory
2575 Sand Hill Road Menlo Park, California 94025
